# Immune-Mediated Organ-Specific Reactions to COVID-19 Vaccines: A Retrospective Descriptive Study

**DOI:** 10.3390/ph16050720

**Published:** 2023-05-09

**Authors:** Carmen Ruiz-Fernández, Ricardo Cuesta, Susana Martín-López, Javier Guijarro, Arturo López Gómez de las Huertas, Mikel Urroz, Laura Miguel-Berenguel, Miguel González-Muñoz, Elena Ramírez

**Affiliations:** 1 Immunology Department, La Paz University Hospital-IdiPAZ, 28046 Madrid, Spain; carmen.ruiz@idipaz.es (C.R.-F.); ricardo.cuesta@salud.madrid.org (R.C.); lmiguelb@salud.madrid.org (L.M.-B.); 2 Clinical Pharmacology Department, La Paz University Hospital-IdiPAZ, Faculty of Medicine, Universidad Autónoma de Madrid, 28046 Madrid, Spain; smartinlopez@salud.madrid.org (S.M.-L.); franciscojavier.guijarro@salud.madrid.org (J.G.); aglopezhuertas@salud.madrid.org (A.L.G.d.l.H.); mikel.urroz@salud.madrid.org (M.U.)

**Keywords:** COVID-19 vaccines, adverse drug reactions, delayed hypersensitivity reactions, causality algorithms, lymphocyte transformation test

## Abstract

Severe acute respiratory syndrome coronavirus 2 caused the global COVID-19 pandemic and public health crisis, and it led to the rapid development of COVID-19 vaccines, which can cause rare and typically mild hypersensitivity reactions (HRs). Delayed HRs to COVID-19 vaccines have been reported, and the excipients polyethylene glycol (PEG)2000 and polysorbate 80 (P80) are the suspected culprits. Skin patch tests do not help in diagnosing delayed reactions. We aimed to perform lymphocyte transformation tests (LTT) with PEG2000 and P80 in 23 patients with suspected delayed HRs. Neurological reactions (n = 10) and myopericarditis reactions (n = 6) were the most frequent complications. Seventy-eight percent (18/23) of the study patients were admitted to a hospital ward, and the median time to discharge was 5.5 (IQR, 3–8) days. Some 73.9% of the patients returned to baseline condition after 25 (IQR, 3–80) days. LTT was positive in 8/23 patients (5/10 neurological reactions, 2/4 hepatitis reactions and 1/2 rheumatologic reactions). All myopericarditis cases had a negative LTT. These preliminary results indicate that LTT with PEGs and polysorbates is a useful tool for identifying excipients as causal agents in HRs to COVID-19 vaccines and can play an important role in risk stratification in patients with HRs.

## 1. Introduction

Severe acute respiratory syndrome coronavirus 2 (SARS-CoV-2) caused the global COVID-19 pandemic and public health crisis, leading to the rapid development of SARS-CoV-2 vaccines, some of which were globally administered after authorization [1]. Different platforms were used to develop COVID-19 vaccines, which are based on nucleic acid, recombinant viruses, virus protein subunits and inactivated viruses. The first vaccines approved were based on nucleic acid and recombinant viruses. Pfizer/BioNtech and Moderna used an innovative platform based on messenger ribonucleic acid (mRNA) encoding the spike protein, and Johnson & Johnson and Oxford/AstraZeneca developed recombinant viruses with coronavirus DNA coding for the spike protein on non-replicating adenovirus vectors. To date, over twenty vaccines have been granted approval for emergency use. Among these vaccines, the European Commission has granted eight conditional marketing authorizations for vaccines developed by BioNTech and Pfizer, Moderna, AstraZeneca, Janssen Pharmaceutica NV, Novavax, Valneva, Sanofi and GSK and HIPRA, following positive results from evaluations of their safety and efficacy carried out by the European Medicines Agency.

COVID-19 vaccines are safe and effective, and severe reactions after vaccination are rare. Among the most common reported side effects are fatigue, fever, headache, myalgia and pain and/or redness at the injection site, with mild or moderate symptoms. However, despite the benefits of COVID-19 vaccination, some rare adverse effects have been associated with the use of the vaccines developed against SARS-CoV-2, especially those based on mRNA and non-replicating viral vector technology. Rare adverse events reported include allergic and anaphylactic reactions, thrombosis and thrombocytopenia, myocarditis, Bell’s palsy, transient myelitis, Guillain–Barré syndrome, recurrences of herpes-zoster, autoimmunity flares, epilepsy and tachycardia [2].

A number of these vaccines contain new ingredients that had not previously been employed in vaccine manufacturing. The mRNA encoding the spike protein is encapsulated in lipid nanoparticles containing lipids and polyethylene glycol (PEG) 2000, which differs from the PEG used in other vaccines and healthcare products and serves as a stabilizer to prevent rapid enzymatic degradation of mRNA and facilitate in vivo delivery in the Pfizer-BioNtech and Moderna vaccines. Moderna’s vaccine also contains trometamol as an excipient, whereas AstraZeneca’s and Johnson & Johnson’s vaccines contain polysorbate 80 (P80), a nonionic surfactant and emulsifier often used in foods and cosmetics. AstraZeneca’s vaccine also contains ethylenediaminetetraacetic acid (EDTA) [3,4].

Anaphylaxis and other allergic reactions were reported shortly after the vaccination campaigns began, impelling various international public health agencies and allergy-related organizations to provide anaphylaxis management recommendations and evidence-based guidelines for vaccinating patients with allergies [5,6,7].

As with other drugs, vaccines can produce hypersensitivity reactions (HRs) that can be caused by the active ingredient or excipients. These HRs are rare and usually mild. Although the cause of HRs to COVID-19 vaccines has yet to be determined, PEG 2000 in mRNA vaccines and P80 in viral vector vaccines are the suspected culprits [8,9,10]. This suspicion is based on the demonstrated involvement of PEG and P80 in immediate and delayed HRs to drugs [11,12,13,14,15]. With regard to immediate HRs, there are now multiple reports of individuals with previous episodes of PEG anaphylaxis, with skin test results positive to both PEG and P80 or immediate reactions to pegylated drugs who have tolerated either the mRNA or adenoviral vector COVID-19 vaccines [16,17].

Various local and systemic delayed HRs to COVID-19 vaccines have been reported. Among the HRs, delayed cutaneous reactions and myocarditis have been more frequently reported with mRNA vaccines, while thromboembolic events and thrombosis with thrombocytopenia and neurological reactions have been associated with adenovirus vector vaccines [18,19]. The vaccine component(s) responsible for these HRs and the underlying mechanisms remain to be elucidated. Skin patch tests with excipients do not help diagnose delayed reactions [20,21], and, as far as we know, there has been no study to date on the contribution of lymphocyte transformation tests with the excipients of COVID-19 vaccines to diagnosing delayed HRs.

We conducted a descriptive retrospective study to analyse the cellular immune response to PEG2000 and P80 using the lymphocyte transformation test in patients with suspected delayed HRs to COVID-19 vaccines.

## 2. Results

A total of 23 patients with suspected delayed HRs after being administered COVID-19 vaccines were referred to the Pharmacovigilance Unit to assess the possibility that the vaccine was responsible for the HRs (Table 1). The patients’ median age was 36.0 (IQR, 28.0–59.0) years, and seven were women. With regard to the clinical entities, 10 cases of neurological reactions, 6 myopericarditis reactions, 4 hepatitis reactions, 2 rheumatologic reactions and 1 severe skin reaction were observed. Adverse events occurred after receiving the BNT162b2 (n = 16; 7 at the first dose, 7 at the second dose and 2 at the third dose), mRNA-1273 (n = 3; 2 at the first dose and 1 at the second dose) and the ChAdOx1–S (n = 4; 3 at the first dose, 1 at the first and second doses) vaccines. Immune responses to the vaccines were analysed in 21/23 patients. All patients but two (P3 and P20) showed cellular immune responses, three of them (P1, P13 and P16) had previously had COVID-19 and P4 and P8 reported a previous history of adverse drug reactions. The median latency (time from vaccination to event) was 4 (IQR, 2–10) days. Seventy-eight percent (18/23) of the patients were admitted to the hospital ward, and the median time to discharge was 5.5 (IQR, 3–8) days. Some 73.9% of the patients returned to baseline condition (clinical histories revised in February 2023) after 25 (IQR, 3–80) days of follow-up. Only two patients (P1 and P21) were administered a new dose after HRs. P1 was administered the same vaccine, while P21 was administered a heterologous vaccine; neither patient showed an adverse drug reaction. The remaining patients were not vaccinated with new doses after the HRs because they had had COVID-19 before the reaction, they had been administered the full schedule or they refused to be revaccinated (Table 1). None of the patients had previously experienced HRs to PEG or polysorbates or other vaccines.

The causality algorithms yielded a median score of +6 (IQR, +5–+7) for the COVID-19 vaccines (Table 2). Six reactions were classified as possible, 16 probable and 1 definite. To assess LTT performance in these cases, we first assayed for the toxicity of the excipient concentration curve in three controls and subsequently performed the test with 17 vaccinated controls. Mean (±SD) SIs of 1.5 ± 0.7 and 1.4 ± 0.8 were found with PEG 2000 and P80, respectively. An SI positivity threshold of 3 was determined as the mean (+2 SD) SI in the controls. According to this cut-off, the LTT with excipients was positive in 8/23 patients (34.8%), 5 with both PEG2000 and P80, 2 with PEG2000 and 1 with P80. The distribution of positive LTT results by adverse reaction was 5/10 neurological reactions, 2/4 hepatitis reactions and 1/2 rheumatologic reactions. The median SIs in the positive LTTs were 4.1 (IQR, 3.4–11.7) and 4.4 (IQR, 3.7–7.5) for PEG2000 and P80, respectively (Table 2). The median interval between the onset of the reaction and the study was 4 (IQR, 3–7) months. LTT was also performed with concomitant drugs, which scored ≥3 (P1 and P14), and negative results (SI < 2) were obtained (Table 2).

**Figure 1:**
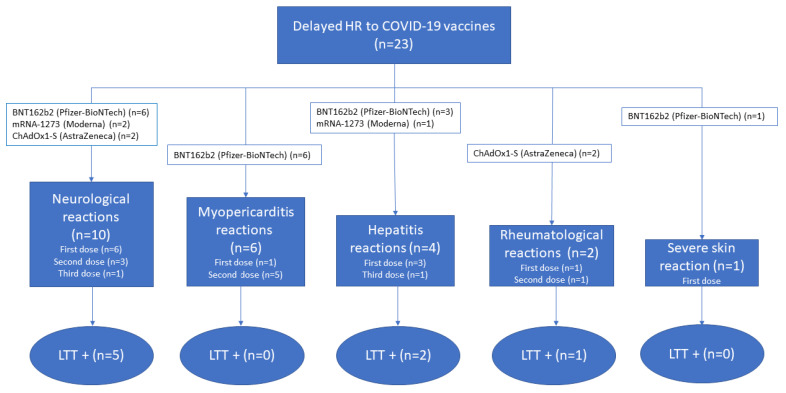
Summary diagram of the main findings of this study. HR, hypersensitivity reactions. LTT, lymphocyte transformation test.

There was the possibility of performing a second LTT in four patients (Table 2). In three of them, the second LTT was performed more than a year after the HR. We observed that the cellular immune response to excipients became negative. The main findings of the present study are summarized in Figure 1.

## 3. Discussion

The mRNA vaccines developed by Pfizer-BioNtech and Moderna use a lipid-based nanoparticle carrier system that prevents the rapid enzymatic degradation of mRNA and facilitates in vivo delivery. This nanoparticle is stabilized by a PEG 2000 lipid conjugate that provides a hydrophilic layer, prolonging the vaccine’s half-life. These ingredients had not been previously employed in vaccine manufacturing, and the experience with adverse effects and risk of allergic reactions is, therefore, limited [5]. There have already been reports of patients who have experienced immediate or delayed HRs to COVID-19 vaccines [19,21,22].

The mechanism behind the immediate reactions remains elusive, and current evidence supports a non-IgE mechanism. A systematic review and meta-analysis of case studies and case reports of immediate allergic reactions to the first vaccine dose have shown that most individuals tolerated subsequent doses, and only <0.5% of the patients developed severe immediate allergic reactions [23].

Various local and systemic delayed HRs to COVID-19 vaccines have been reported, most of them as benign rashes. However, cases of acute, generalized, exanthematous pustulosis, erythema multiforme and other blistering rashes have also been reported [17]. Our case series of suspected delayed HRs to COVID-19 vaccines included a case of erythema multiforme and other systemic reactions that have already been reported [18,21,24,25,26,27,28].

Causality assessment of identified adverse events during drug exposure is crucial due to its implications for patients and the risk–benefit ratio evaluations of medicines. The WHO global introspection method, despite its usefulness, has been subject to criticisms of subjectivity and imprecision since it is mainly based on expert clinical judgments [29,30]. Since 1977, several decisional algorithms have been published, which, by combining and scoring different criteria as an explicit approach, have claimed the advantage of avoiding subjective bias. These algorithms have high (nearly 100%) sensitivity and positive predictive value [31]. To assess the likelihood of COVID-19 vaccines being involved in HRs, causality algorithms were used once other possible causes of the HRs were reasonably ruled out. Causality algorithms can help in identifying the causative drug, especially in cases with concomitant drug use [32,33]. In addition, they can be performed from the first moment of suspicion of an adverse drug reaction. However, they have low (not higher than 37.5%) specificity and negative predictive values [31]. Together with algorithms, the LTT constitutes a useful tool for assisting in the diagnosis of adverse drug reactions [34].

A previous study assessed the utility of lymphocyte activation tests in investigating the cellular immune response to pegylated drugs [35]. Our results show that a number of patients reporting delayed HRs to COVID-19 vaccines are sensitized to their excipients. Most of the patients experienced HRs after the first vaccine dose. However, given that PEGs and polysorbates are widely used, these patients might be previously sensitized to these compounds [11]. Histologic evaluation has shown a type IV hypersensitivity mechanism in delayed major local reactions to mRNA vaccines [36,37], and the LTT results would also support a type IV hypersensitivity mechanism in systemic reactions. We observed three patients who were monosensitized to the vaccine excipient to which they were exposed when vaccinated, while the remaining patients showed reactivity to PEG2000 and PS80. Cross-reactivity between PEG and polysorbate has been suggested due to their similar chemical structures. The clinical relevance of cross-reactivity between PEG and PS80 has been observed in very few cases of immediate HR to PEG [12,38]. However, clinical cross-reactivity has not been reported in the COVID-19 vaccination setting. It has been reported that patients with confirmed immediate PEG allergy tolerated the PS80-containing AstraZeneca COVID-19 vaccine [16]. Given that our patients had not been administrated new vaccine doses, we cannot provide data on the clinical significance of the cross-reactivity observed in vitro.

Neurological complications have been reported as rare adverse reactions to COVID-19 vaccines [39] in the context of the immune response to vaccines. These complications include Guillain–Barré syndrome, an acute severe acquired immune-mediated inflammatory polyradiculoneuropathy that affects peripheral nerves. Although the pathogenic mechanism is not fully understood, it has been associated with several infectious agents including SARS-CoV-2. A number of cases with this syndrome have also been reported following COVID-19 vaccination [39]. Given that spike proteins bind to gangliosides [40], cross-reacting anti-spike antibodies generated by vaccines could be involved in the onset of Guillain–Barré syndrome. Other neurological reactions such as palsies have been hypothesized to be related to the disruption of immunological tolerance to myelin sheath antigen induced by vaccines [39]. Fifty percent of cases of neurological complications in the present study showed type IV hypersensitivity to excipients, suggesting that it could be considered as another immune-mediated mechanism playing a role in the pathogenesis of these adverse reactions.

Interestingly, none of the six patients with myocarditis or myopericarditis showed a positive LTT. Persistently elevated levels of freely circulating, unbound by antibodies, full-length spike proteins have been reported up to 3 weeks after vaccination in patients with myocarditis [41]. The fact that most cases of myocarditis occur after the second dose raises the possibility of dose accumulation if the mRNA persisted [42]. As in other series [43], our patients with myocarditis showed no delayed HRs (such as serum sickness, eosinophilic myocarditis, thromboembolic events, thrombocytopenia, cytokine storm and hemophagocytic lymphohistiocytosis) as a cause of myocarditis after vaccination with COVID-19 mRNA.

The main limitation of this study is the small sample size, it is also a retrospective study; therefore the results are only preliminary. Further research with larger sample sizes and analysis of the tolerance to new vaccine doses would define the clinical relevance of this in vitro test.

The study’s main preliminary conclusion is that LTT with PEGs and polysorbates is a useful tool for identifying excipients as the causal agents in HRs to COVID-19 vaccines and can play an important role in risk stratification in patients with HRs, given that many such vaccines and other drugs contain these excipients.

## 4. Materials and Methods

### 4.1. Patients

Patients with symptoms compatible with delayed HRs to COVID-19 vaccines (occurring more than four hours after vaccination) between March 2021 and September 2022 were included in the study. All information was retrospectively collected from electronic medical records. After their clinical evaluation, the patients were referred to the Pharmacovigilance Unit of the Clinical Pharmacology Department (La Paz University Hospital, Madrid, Spain) to assess whether there was a causal relationship between the symptoms and vaccines. Probability scores were assigned using the Spanish pharmacovigilance system (SPVS) algorithm. The SPVS algorithm is a modification of the Karch and Lasagna algorithm [44]; it can be applied to the causality assessment of adverse effects in Spanish-speaking countries [45]. The Roussel Uclaf causality assessment method (RUCAM) algorithm was used for the causality evaluation in cases of hepatitis [46].

This study was approved by the La Paz University Hospital Ethics Committee. Due to the study’s retrospective nature, the absence of informed consent was allowed. For all patients initially categorized as having suspected delayed HRs, a complete report was submitted to the pharmacovigilance centre in Madrid, Spain https://www.notificaram.es (accessed on 30 September 2022).

The inclusion criteria were all cases with suspected delayed HRs to COVID-19 vaccines based on clinical history, ruling out alternative causes and applying causality algorithms to evaluate the causal relationship of the vaccine to the adverse reaction. Cases were excluded when a disease or medications other than COVID-19 vaccines were more likely the cause of the side effect.

### 4.2. Cellular Immune Response to COVID-19 Vaccines

Heparinized whole blood was drawn into collection tubes containing epitopes derived from the S1 subunit of the S protein, activating T CD4+ cells or epitopes from the S1 and S2 subunits of the S protein, inducing both T CD4+ and CD8+ cell activation (QuantiFERON^®^ Human IFN-γ SARS-CoV-2, Qiagen, Madrid, Spain). After incubation at 37 °C for 16–24 h and centrifugation, plasma IFN-γ (IU/mL) was measured using an enzyme-linked immunoassay (QuantiFERON^®^ Human IFN-γ SARS-CoV-2, Qiagen). Samples were considered positive for T-cell response when exceeding the cut-off value of 0.015 IU/mL [47].

### 4.3. Lymphocyte Transformation Test

LTT was performed using several concentrations of the excipients PEG 2000 (CAS 25322-68-3) and P80 (CAS 9005-65-6; ThermoFisher Scientific, Madrid, Spain) in the patients and asymptomatic vaccinated controls. LTT was performed after event recovery and at least 1 month after steroid therapy was stopped, if applicable. Lymphocyte proliferation was measured as previously described [34,48]. Mononuclear cells were separated over a density gradient (Histopaque–1077, Sigma-Aldrich, Madrid, Spain) from fresh peripheral blood and were plated in flat bottom wells of microtiter plates at 2 × 10^5^ cells/well. Cells were incubated for 6 days with various excipient concentrations (10 µg/mL–0.01 µg/mL) in triplicate. We used phytohemagglutinin (PHA, 5 μg/mL) as a positive control. The excipient concentration curve was previously assayed for toxicity, adding the excipients to PHA-stimulated cell cultures from three controls. For the final 18 h of the incubation period, proliferation was determined by adding 1 μCi [^3^H] of thymidine. Proliferative responses were calculated as the stimulation index (SI), defined as the ratio between the mean values of the counts per minute in cultures with the drug and those obtained without the drug. LTT was also performed in 17 vaccinated participants who did not experience adverse events after vaccination. An LTT result was considered positive when the SI exceeded the threshold (mean of SI + 2 SD of controls) for at least one drug concentration. The patients were considered as having immune-mediated HR when at least one LTT was positive.

### 4.4. Causality Assessment

The causality assessment was performed using the SPVS algorithm [45] and updated RUCAM for hepatitis cases [46]. These algorithms score causality according to various questions related to the temporal relationship between exposure to a drug and the liver injury, the exclusion of an alternative non-drug-related cause, the effect of the drug in the event of re-exposure, evidence in the literature regarding the relationship between the drug and the event, withdrawal effect, patient risk factors for the adverse event and exposure to other drugs that could also explain the event. The final score classifies the drug into five categories: unrelated (<0), conditional (1–3), possible (4–5), probable (6–7) and definite (≥8) according to the SPVS algorithm or highly probable (≥9), probable (6–8), possible (3–5), unlikely (1–2) or excluded (<0) as per RUCAM. The COVID-19 vaccine was considered related to the induction of the adverse reaction when it scored ≥4 in the SPVS and ≥3 in the RUCAM.

### 4.5. Statistical Analysis

Continuous variables are expressed as mean and standard deviation (SD) or median and interquartile range (IQR) according to the Kolmogorov–Smirnov normality test. Categorical variables are expressed in absolute terms and percentages. The data were analysed using IBM SPSS Statistics version 21.0 (IBM Corporation, Armonk, NY, USA). Given the descriptive preliminary nature of the study, a sample size calculation was not performed.

## Figures and Tables

**Table 1:** Patients’ demographics and clinical characteristics.

Patient	Sex	Age (Years)	Underlying Disease	Previous COVID-19/Previous ADR	Vaccine (Dose at Which Reaction Occurs)	Cellular Immune Response to Vaccine	Adverse Reaction	Latency	Outcome/Time until Total Recovery	Tolerance to Subsequent COVID-19 Vaccination
P1	M	30	Allergic rhinitis and asthma	y/n	BNT162b2 (Pfizer-BioNTech) (1)	Positive	Mixed hepatitis	7 days	Admitted to hospital ward for 4 days/98 days	Yes (mRNA-1273)
P2	F	15	No	n/n	BNT162b2 (Pfizer-BioNTech) (1)	Positive	Hepatocellular hepatitis	4 days	Discharged from ED/13 days	NV
P3	M	22	No	n/n	mRNA-1273 (Moderna) (1)	Negative	Left hypoglossal nerve paresis	6 days	Admitted to hospital ward for 8 days/63 days	NV
P4	M	15	No	n/y (sulphonamide)	BNT162b2 (Pfizer-BioNTech) (2)	Positive	Myopericarditis	4 days	Admitted to hospital ward for 7 days/32 days	NV
P5	M	36	Dyslipidaemia	n/n	BNT162b2 (Pfizer-BioNTech) (1)	Positive	Myocarditis	15 days	Admitted to hospital ward for 3 days/3 days	NV
P6	M	16	Subclinical hypothyroidism	n/n	BNT162b2 (Pfizer-BioNTech) (2)	Positive	Myopericarditis	4 days	Admitted to hospital ward for 3 days/3 days	NV
P7	F	67	Hypothyroidism, dyslipidaemia	n/n	ChAdOx1-S (AstraZeneca) (1)	Positive	Cranial nerves III and IV neuropathy	2 days	Admitted to hospital ward for 4 days/54 days	NV
P8	F	56	Graves’ disease	n/y (acetylsalicylic acid)	mRNA-1273 (Moderna) (1)	ND	Hepatocellular hepatitis	7 days	Admitted to hospital ward for 14 days/104 days	NV
P9	M	73	Depression, essential tremor	n/n	BNT162b2 (Pfizer-BioNTech) (2)	Positive	Pleuropericarditis	15 days	Admitted to hospital ward for 8 days/25 days	NV
P10	F	28	No	n/n	BNT162b2 (Pfizer-BioNTech) (1)	Positive	Aseptic meningitis	1 day	Admitted to hospital ward for 5 days/27 days	NV
P11	F	32	Graves’ disease, asthma	n/n	ChAdOx1-S (AstraZeneca) (1)	Positive	Fever, arthralgia, myalgia, general discomfort	3 days	Seen in outpatient care/No resolution yet *	NV
P12	M	38	Multiple sclerosis	n/n	BNT162b2 (Pfizer-BioNTech) (2)	Positive	Myocarditis	4 days	Admitted to hospital ward for 2 days/1 day	NV
P13	M	28	No	y/n	BNT162b2 (Pfizer-BioNTech) (1)	Positive	Erythema multiforme	10 days	Admitted to hospital ward for 6 days/24 days	NV
P14	F	76	Dyslipidaemia, hypertension	n/n	BNT162b2 (Pfizer-BioNTech) (3)	Positive	Mixed hepatitis	7 days	Discharged from ED after 2 days of observation/120 days	NV
P15	M	59	Hyperlipidaemia	n/n	BNT162b2 (Pfizer-BioNTech) (2)	Positive	Guillain–Barré syndrome	3 days	Admitted to hospital ward for 2 days/No resolution yet *	NV
P16	M	43	Dyslipidaemia	y/n	mRNA-1273 (Moderna) (2)	Positive	Acute transient encephalopathy	1 day	Discharged from ED after 1 day of observation/1 day	NV
P17	M	64	Hypertension, asthma	n/n	ChAdOx1-S (AstraZeneca) (1 and 2)	Positive	Polyarthralgia (first dose) Polyserositis (second dose)	13 days	First dose: Discharged from ED Second dose: Admitted to hospital ward for 7 days/First dose: 41 days Second dose: 17 days	No vaccination after second AR
P18	F	31	No	n/n	BNT162b2 (Pfizer-BioNTech) (1)	Positive	Paraesthesia in right arm and foot	4 h	Seen in outpatient care/Unknown	NV
P19	M	33	No	n/n	BNT162b2 (Pfizer-BioNTech) (2)	Positive	Myocarditis	1 day	Admitted to hospital ward for 3 days/3 days	NV
P20	M	51	Urinary tract infection	n/n	BNT162b2 (Pfizer-BioNTech) (1)	Negative	Guillain–Barré syndrome	2 days	Admitted to hospital ward for 46 days/No resolution yet	NV
P21	M	68	Benign prostatic hyperplasia	n/n	ChAdOx1-S (AstraZeneca) (1)	Positive	Guillain–Barré syndrome	36 days	Admitted to hospital ward for 34 days/360 days	Yes (BNT162b2)
P22	M	32	No	n/n	BNT162b2 (Pfizer-BioNTech) (2)	ND	Guillain–Barré syndrome	1 day	Admitted to hospital ward for 9 days/No resolution yet *	NV
P23	M	53	Hypertension, obesity	n/n	BNT162b2 (Pfizer-BioNTech) (3)	Positive	Paraesthesia (second dose) Aggravation of paraesthesia (third dose)	20 days from second dose	Admitted to hospital ward for 1 day/No resolution yet *	NV

* Revised in February 2023. ADR, adverse drug reaction. NV, not vaccinated after adverse drug reaction. ND, not determined.

**Table 2:** Evaluation of the adverse drug reactions to COVID-19 vaccines.

Patient	Algorithm Score	Concomitant drugs (Algorithm Score/Stimulation Index)	Time to Analysis (Months)	Stimulation Index PEG 2000	Stimulation Index P80
P1	+6	Bilastine (+3/1.5) Terbutaline (+1/not performed)	3	**4.1**	2.3
P2	+7	No	1.5	**3.4**	**4.3**
P3	+6	No	3.5	0.5	0.4
P4	+6	No	2	0.8	1.3
P5	+7	No	4	0.4	0.7
P6	+6	No	3	0.3	0.5
P7 *	+7	No	7	**12.1**	**4.6**
			19	1.6	1.7
P8	+7	No	6	1.8	1
P9	+7	Tramadol (−1/not performed)	7	0.7	0.9
P10	+6	No	4	1.4	0.4
P11 *	+4	No	2.5	1.3	**3.6**
			9	**9.3**	**12.6**
P12	+7	No	5	0.9	1.2
P13	+5	No	6	2.3	2.8
P14	+7	Paracetamol (+5/0.9)	1.5	0.8	0.8
P15 *	+4	No	8	**10.7**	**6.4**
			17	**3.3**	1
P16	+5	No	3	1.1	1.3
P17	+8	No	8	1.7	1.3
P18 *	+6	No	7	**11.7**	2.8
			13	2.7	ND
P19	+6	No	3	1	1.3
P20	+4	No	12	1.6	1.7
P21	+6	Tadalafil, Silodosin (−1/not performed)	10	1.1	1.4
P22	+4	No	1	**3.8**	**10.9**
P23	+7	Enalapril/Hydrochlorothiazide (+1/not performed)	8	**3**	**3.7**

* Patients with two lymphocyte transformation tests. The bold numbers mean positive stimulation index of the lymphocyte transformation tests.

## Data Availability

Data are contained within the article.
